# Exploring Digital Stress of Finnish Adolescents and Their Parents

**DOI:** 10.3390/children11121472

**Published:** 2024-11-30

**Authors:** Dimitrios Siakas, Niilo Siakas, Kerstin Siakas, Georgios Lampropoulos

**Affiliations:** 1 Häme University of Applied Sciences, 13100 Hämeenlinna, Finland; dimitrios.siakas@gmail.com; 2 Vaasan Lyseon Lukio, 65100 Vaasa, Finland; niilo.siakas@gmail.com; 3 Department of Information and Electronic Engineering, International Hellenic University, 57400 Sindos, Greece; 4 Department of Production—Industrial Management, University of Vaasa, 65200 Vaasa, Finland; 5 Department of Applied Informatics, University of Macedonia, 54636 Thessaloniki, Greece; glampropoulos@uom.edu.gr; 6 Department of Education, University of Nicosia, 2417 Nicosia, Cyprus

**Keywords:** digital stress, social media, digital social multitasking, DSMT, burnout, case study, health, psychology, education

## Abstract

Background/Objectives: The way people communicate has significantly changed due to technological advances and penetration of cell phones and broadband connection into everyday activities. Nowadays, individuals are constantly connected through the Internet. This influences social experiences, self and social identity, and can cause digital stress, which often leads to negative emotions, psychosomatic outcomes, and diseases. It is a matter of concern for the adolescents and their families. This paper investigates how Information and Communication Technologies (ICTs) and social media influence adolescents’ and parents’ digital stress and anxiety. Methods: Based on two digital stressors scales, a 30-item questionnaire was created. A quantitative analysis of data deriving from 164 Finnish adolescents and 53 of their parents regarding digital stress was conducted. Results: “Privacy Intrusion”, “Availability stress/Online vigilance”, and “Usefulness” were the most essential stressor factors for both students and parents. Additionally, the most significant stressor factors for students were the “Fear of Missing Out/FoMO” and “Approval Anxiety” factors while for parents were the “Safety/Security” and “Technical Support” factors. No significant differences were observed between the overall digital stress of adolescents and parents. In contrast to the existing literature, no connection between “Connection (Information) Overload” and digital stress was found. Conclusions: This paper contributes to debates regarding the effects of the use of digital resources, ICTs, and social media on processes at school, at work, and at home. The findings of our study confirm and further validate those of previous studies with the exception of “Connection (Information) Overload”, which needs to be further investigated to understand the reasons behind it.

## 1. Introduction

People feel stressed when “*demands exceed available coping resource*” [1] (p. 63). Digital stress is defined as “*stress resulting from a strong and perhaps almost permanent use of information and communication technology (ICT) that is triggered by permanent access to an inconceivable amount and diversity of (social) content*” [2] (p. 237). The experienced level of digital stress is formed by how we feel we can cope in a certain context [3]. Digital stress has also been used in relation to social media to signify cognitive, emotional, and physical excitement [4,5]. Social media use is considered to have an impact on psychosocial outcomes developed by digital stress [6,7]. The meaning of being social has changed. Reinecke et al. [8] emphasized that digital stress is a comprehensive term describing diverse stress phenomena occurring due to certain features of social media [8]. Digital stress can be distinguished into stimulus to stress, also called stressors, and reactions to stress [9]. Stressors can be categorized into (i) chronic stress that can develop if stress occurs consistently and repeatedly, (ii) stressful events happening in normal life situations, such as illness or death and (iii) minor daily life hassles, which are usually quickly solved [10]. Reactions to stressful situations can be physical (e.g., headache), emotional (e.g., crying), or behavioral (e.g., aggression towards others), and they can occur in a relatively short period of time (mood disturbances) or over a long period of time (depressive symptoms) [9]. A rather radical change has taken place in the last decade in how people communicate with each other. The relationship between the use of ICTs, in particular digital media, and psychological outcomes is considered a complicated phenomenon [3]. Apprehensions regarding extended use of social media and potential undesirable impacts on psychological well-being are increasingly expressed [11]. Nick et al. [12] associated digital stress with later increases in depressive symptoms.

Mobile devices and broadband connection offer the option to connect to peers almost whenever and wherever and stay updated and informed. Digital connectedness with friends and family through social media is beneficial when there is a significant distance between users. A “*feeling of belonging*” and a “*feeling of immediate social support*” are created because of the possibility of being in touch and connected via the Internet [13]. However, many shortcomings can also be identified. For example, social networks, a popular communication means for adolescents, have both positive (e.g., sharing context with study mates and reconnecting with old friends) and negative (e.g., fear of cyberbullying and pseudonym) impacts and consequences. The results of Nick et al. [12] highlighted that digital stress correlates with the increased popularity of using social media, the level of importance of peers, and diverse mental health issues. Symptoms of depression originating in digital stress were found to be longitudinally increasing. The multitude of information available on the internet creates an information overload since we constantly need to make decisions regarding what to read, watch, listen to, and what to follow. Similarly, the constant communication demand may result in (social) information overload and stress. In addition, the everlasting access to information both provokes and facilitates continuous switching of tasks and multitasking. Both adolescents and adults, in particular within developed countries, appear to live in a continuously online world, where everything is connected and the online identity is closely linked to mental health and well-being referring to real life [14]. Some theoretical and empirical studies regarding the digital stress and well-being of adolescents have been conducted [15]. Nick et al. [12] confirmed that digital stress originating in social media behaviors is understudied in adolescents. Research regarding the impacts and influences of digital stress on students’ well-being in educational contexts, is relatively scarce. This study aims to measure the effects of ICTs and social media on the level of digital stress of Finnish adolescents and their parents. Despite the fact that the issue of stress and anxiety produced by ICTs and social networking are widely studied topics at the international level [16,17,18], the originality of this article lies in establishing a study among students and parents jointly.

The remainder of this paper is organized as follows: In Section 2, it goes over other related studies and in Section 3, it presents the research method adopted. Section 4 demonstrates the results of our survey regarding digital stress and digital mental overload. Section 5 includes the conclusions and points out potential future research perspectives.

## 2. Related Work

A conceptual model of stress factors associated with digital stress was developed by Steele et al. [3] and the main grouping of the factors are as follows [3] (p. 18):

- Connection (information) overload: “*Distress resulting from the subjective experience of receiving excessive input from digital sources, including notifications, text messages, posts, etc.*”.
- Availability Stress: “*Distress (including guilt and anxiety) resulting from beliefs about others’ expectations that the individual respond and be available by digital means*”.
- Fear of Missing Out (FoMO): “*Distress resulting from the real, perceived, or anticipated social consequences of others engaging in rewarding experiences from which one is absent*”.
- Approval Anxiety: “*Uncertainty and anxiety about others’ responses and reactions to one’s posts or to elements of one’s digital footprint*”.

Khetawat and Steele [15] further confirmed the four digital stress components introduced by Steele et al. [3] but also included online vigilance as an additional factor. Specifically, online vigilance is defined as “*users’ permanent cognitive orientation towards online content and communication as well as their disposition to exploit these options constantly*” [19] (p. 1). Le Roux and Parry [20] defined online vigilance as attention to online connectedness-related signals and prioritization of subsequent communication. Based on the stress components that Steele et al. [3] identified, we present related work in a similar sequence. Taking the nature of this study into account, the term information overload was used instead of connection overload.

### 2.1. Information Overload

According to Bawden and Robinson [21] (p. 5), information overload is described as “*information overabundance, infobesity, infoglut, data smog, information pollution, information fatigue, social media fatigue, social media overload, information anxiety, library anxiety, infostress, infoxication, reading overload, communication overload, cognitive overload, information violence, and information assault*”. The growth of information and its transmission in diverse forms on various digital channels causes new challenges for individuals who try to cope with simultaneous communication demands from multiple sources. A high individual perception of the volume of incoming social information and the perceived possibility of not being able to cope with it, create connection overload. When the demands from peers and the environment exceed the perceived processing capacity, perceived information overload takes place [22]. However, Belabbes et al. [23] emphasized that there is no commonly accepted agreement regarding the term information overload. For example, Sweller [24] named information overload “cognitive overload”, while Misra and Stokols [25] referred to it as “cyber-based overload” which is a sensation of being drowned by large volumes of digital messages on diverse channels with perceived responsibility to keep track of the information and respond to the messages. In order to manage all this information, multitasking is practiced. Belabbes et al. [23] identified triggers, which included (i) the cognitive state of the individual, (ii) poorly defined information needs, (iii) information characteristics (intrinsic including quality and diversity and extrinsic including quantity, speed and relevance), (iv) the working environment, and (v) the space in which the information is dealt with. They also emphasized that information overload reveals itself emotionally (mental health of the user) and cognitively (limitations of individuals). The outcomes of information overload can be divided into internal (impact on the individual) and external consequences on the surrounding environment, such as poor decision making, poor productivity, poor collaboration, dropout/withdrawal from the task, financial loss, and mistakes costing lives [26]. The results of Matthes et al. [27] showed that the perception of information overload can be considered to be an important prognosis of depression and negative effects on the well-being of individuals in the long run. They concluded that information overload has a considerable impact on the mental health of a wider population. The problem of information overload seems to persist, but increasingly with more diverse challenges, such as (i) an increase in types of information (increase in quantity of information, dismissed information, duplicate information), (ii) personal characteristics (lack of prior experience, individual drawbacks, lack of language proficiency), and (iii) task characteristics (complexity of tasks) [21].

### 2.2. Availability Stress

It is generally considered that social pressure, because of peer expectations of being constantly online, is particularly harmful when self-control is lost and satisfaction of needs is impeded [28]. In their study, Świątek et al. [29] found that self-control mediates interrelationships between excessive smartphone use and social media exhaustion (cognitive, behavioral, emotional, and overall social media exhaustion), which is a natural defensive response, triggered when smartphone users are not able to stop using social media and smartphones for unlimited communication. Moreover, Halfmann and Rieger [28] found that only some of the users perceived social pressure as a burden. Demands for availability have been related to negative feelings about well-being (health, happiness, and prosperity) and interruptions in daily activities and sleeping patterns [30]. Stress, hypertension, anxiety, and worries have also been reported [28].

### 2.3. Fear of Missing out

FoMO is “*a pervasive apprehension that others might be having rewarding experiences from which one is absent*” and “*a desire to stay continually connected with what others are doing*” [31] (p. 1841). In a study focusing on adolescents, a more intensive use of social media was triggered by different emotions; boys were feeling anxiety and girls were feeling depressed [32]. Przybylski et al. [32] proposed that FoMO could assist as an intermediary between level of engagement in social media and diverse well-being factors (e.g., general mood, needs, and life satisfaction). Chotpitayasunondh and Douglas [33] proposed FoMO to be a prognosticator of smartphone addiction. Similarly, Gugushvili et al. [34] argued that FoMO could be considered a key predictor of excessive and challenging use of smartphones. Being accepted by one’s peers and being connected to them is important for adolescents [35]. Hence, social networking sites are crucial for social involvement. Gugushvili et al. [34] emphasized that FoMO and problematic use of smartphones are negatively correlated to well-being indicators.

### 2.4. Approval Anxiety

Nick et al. [12] studied the approval anxiety phenomenon and found correlations to social anxiety, depressive symptoms, and the importance of peers (acceptance, popularity, fear of rejection). Loneliness was also associated with psychosocial problems. The correlation between the need for approval and anxiety is reported by Spielberger and Reheiser [36] who emphasized that anxiety can stem from low self-esteem or feelings of insufficiency regarding interpersonal relationships. They argued that personality characteristics are related to the need for approval by individuals, e.g., extrovert individuals have higher needs for approval. External contingencies in domains considered important (e.g., interpersonal interactions) increase anxiety and tension. Studies have also shown that the need for approval is positively associated with depressive symptoms [37]. Socially anxious individuals usually prefer virtual communication instead of in-person face-to-face communications, because it causes less anxiety due to its distributed character (Fernandez et al. [38]. Multitasking is another contemporary way of performing tasks that is very common among adolescents and also seems to influence digital stress.

After the COVID-19 pandemic, students and employees increasingly use digital means. The statuses “*always on*”, “*always accessible*”, and quick responses appear to be the norm, particularly amongst young people. Hefner and Vordener [2] described three stress-evoking processes, namely:

- Accessibility and reactions to communication demands: Individuals, with high susceptibility to peer pressure, highly agreeable and conscientious were proposed to show higher perceived obligations to respond.
- Continuous communication vigilance: A perpetual alertness to a stimulus over long periods, which prompts multitasking and task switching, both mentally exhausting and stressful.
- Permanently reconsidering and presenting oneself: Likely to decrease self-esteem, reduce levels of self-perceived attractiveness and social competence, feelings of envy and increase self-discrepancies, rumination and depression stress.

Additionally, Hefner and Vordener [2] postulated that users’ personalities and particular situations shape how individuals assess and cope with connection cues and prioritization for potential online communication. Weinstein et al. [39] described additional digital-stress-evoking factors shown in Table 1.

The digital-stress-evoking factors presented in Table 1 were defined as “*Privacy intrusion*” by Fisher et al. [40]. Specifically, “*Privacy intrusion*” is usually a complex tort consisting of four distinct torts, namely public disclosure of private facts, intentional intrusion without consent upon a private state of somebody, false claim publicity and impersonation and appropriation for commercial advantage (e.g., through masking an individual’s identity) [40]. Through their analysis of 6280 comments as responses to 180 digital stress stories, Weinstein et al. [39] found that “*Get Help from others*”, “*Communicate Directly*”, “*Cut Ties*”, and “*Ignore the situation*” are the main advice returned by adolescents. “*Utilize digital solutions*”, including “*reporting content* (e.g., “*as a fake profile!*”), unfriending, blocking, changing passwords, making new personal accounts, or staying off of a particular platform” were less commonly proposed. Despite that “*Get help from others*” was scored as the most important advice by adolescents, other studies, such as [41], indicate that adolescents are reluctant to report cyberbullying to adults because of lack of confidentiality, feeling of powerlessness, victim self-blaming, fear of revenge, child vulnerabilities, fear of losing the relationship if the bully is a friend, and expectations regarding the effectiveness of adult interventions. Wright [42] found that high levels of parental intervention in cyber victimization was most decisive for avoiding depression and anxiety, followed by teacher intervention for Chinese adolescents and friend interventions for American adolescents.

### 2.5. Aims of the Current Study

This work has an exploratory purpose of the phenomenon of digital stress caused by ICTs and social networking experienced through the collection of information through an online questionnaire provided to a Finnish secondary school. A quantitative methodological design was decided to be used to achieve the objective of understanding the digital stress phenomenon by investigating the viewpoints, preferences, opinions, or beliefs of, both students and their parents. In this study, resilience to digital stress or coping strategies are not included in consonance with [43], but the emphasis is on identifying characteristics of digital stress in adolescents.

The following research hypotheses are proposed:

**H1:**  *Peer expectations of being constantly online cause digital stress.*

**H2:**  *Fear of missing out from what peers are doing causes digital stress.*

**H3:**  *Peer approval causes digital stress.*

**H4:**  *Digital demands exceeding coping resources cause digital stress.*

**H5:**  *Blurred boundaries between school and home cause digital stress.*

**H6:**  *Unjustifiable intrusion upon the personal life of another person causes digital stress.*

**H7:**  *Fear of digital safety and security issues causes digital stress.*

**H8:**  *Lack of technical support when needed causes digital stress.*

**H9:**  *Useless and unsuitable digital tools cause digital stress.*

**H10:**  *Unreliability digital information cause digital stress.*

## 3. Research Method

### 3.1. Participants

The data analyzed in this study were derived from a sample of 217 Finnish participants. Specifically, 164 (75.6%) of the participants were secondary school students and 53 (24.4%) were parents of theirs. The average age of the students was 14.5 years old and of parents 43.1. Additionally, not all the students’ parents completed and/or returned the questionnaire; hence, the total number of students is higher than that of parents. No indication to relate a parent to a student was used so that an overall assessment of the participants could be made. As far as the participants’ gender is concerned, the distribution of students was equal as there were 82 female (50.0%) and 82 male (50.0%) students. On the contrary, predominantly female parents (84.9%) completed the questionnaire.

### 3.2. Procedure

The recruitment of the sample was a convenience sample. The authors had personal experience from adolescents with digital stress and wanted to explore it by using a bigger sample including both adolescents and their parents. Hence, the rector of the secondary school was contacted, who agreed with the research team to carry out the study. The questionnaire was first created by the research team English and then translated into Finnish and an online version of the questionnaire was created. The students were provided with the link to the questionnaire by the rector of the secondary school. They were asked to complete the questionnaire on their free time and also to ask their parents to complete it.

### 3.3. Research Instruments

After going over the relevant literature and developing the hypotheses, we built our online survey instrument by extracting suitable questions (with regard to the aims of the study and the research population) from two research instruments, namely:

1. The Digital Stressors Scale (DSS) developed by Fisher et al. [40]. The research instrument measures the perceived stress that results from the use ICTs in the workplace. The DSS entails ten stressor categories and a total of 50 items.
2. The Multidimensional Digital Stressor Scale (MDSS) developed by Hall et al. [6] and Steele et al. [3]. The research instrument measures the perceived stress in college students. The MDSS consists of 5 stressor categories and 24 items.

We created our online questionnaire based on the above two research instruments. In order to have a short and concise version of the questionnaire for promoting higher participant engagement, three items per stressor category were retained, in line with Riedl et al. [44].

The design and construction of the questionnaire based on two scales that have already been developed and validated (Digital Stressors Scale and Multidimensional Digital Stressors Scale) was considered to ensure reliability and enable comparing of results with earlier studies. In order to obtain a clear real-world picture of characteristics, trends and relationships a descriptive and correlational design approach was considered.

As the questionnaire involved closed-ended and Likert-scale questions, descriptive statistics and frequency analysis were used to examine the data. Descriptive statistics, Independent-Sample T-tests, and Analysis of Variance (ANOVA) were used to further explore the data. Additionally, to ensure the internal consistency of the items used, reliability analysis was conducted which revealed a Cronbach’s Alpha value of 0.932 for the 30-main Likert scale items. This fact highlights the high level of internal consistency and overall reliability of the tool used.

To validate the content of the research instrument, two experts validated the questions and the understandability of the Finnish translations.

### 3.4. Measurements

The first part of the questionnaire consisted of demographic questions. Based on the existing literature and the hypotheses, the questions related to digital stress are grouped into 10 stress factors as can be seen in Table 2. Specifically, each of the 10 stress factors is described through 3 questions, which also consider the specific population of this study in terms of age (adolescents and parents), domain (education and workplace), and culture (Finnish). The four first stressor categories and the twelve first questions are extracted from Hall et al. [6]. The following categories and questions are extracted from Fisher et al. [40].

The response rates are on a Likert scale from 1 Strongly disagree to 5 Strongly agree. In addition to the digital stress questions, there were also demographic questions, such as gender, age, field of expertise, working experience in years, time spent daily on computer, tablet and smartphone screens, etc., for enabling categorization of the data.

## 4. Result Analysis

**Figure 1:**
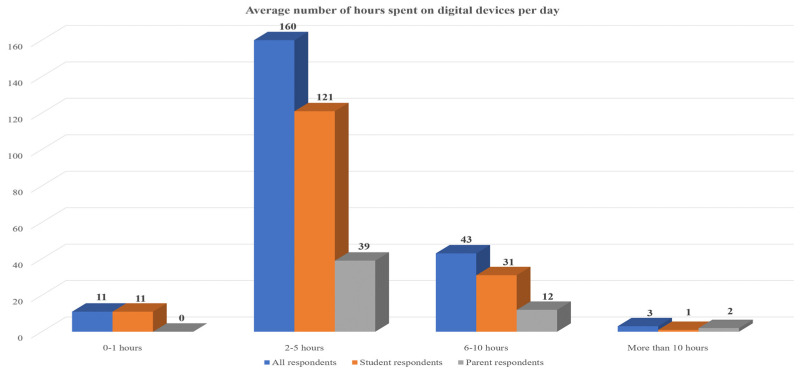
Average number of hours spent on digital devices per day.

As it can be seen in Table 3 and Figure 1, the vast majority of participants (73.7%) spent an average of 2–5 h daily on their computer, tablet, or smartphone, followed by an average of 6–10 h (19%). This is the case for both students and parents. Additionally, no parent reported that they spent less than an hour per day on their digital devices which highlights their need to remain connected and available at all times.

The number of participants who spent more than 10 h on their digital devices daily is low (1.4%) for both students (0.6%) and parents (3.8%). Moreover, parents’ work experience in years was also examined (see Table 4). Particularly, most parents had between 11–20 (34.0%) and 21–30 (32.1%) years of work experience. To a lesser extent, some of the parents had 6–10 (17.0%) and 0–5 (11.3%) work experience. Only a few parents had more than 30 years of work experience (5.7%). This fact, in addition to the parents’ mean age, indicates that most of the parents started working in their early 20s.

**Figure 2:**
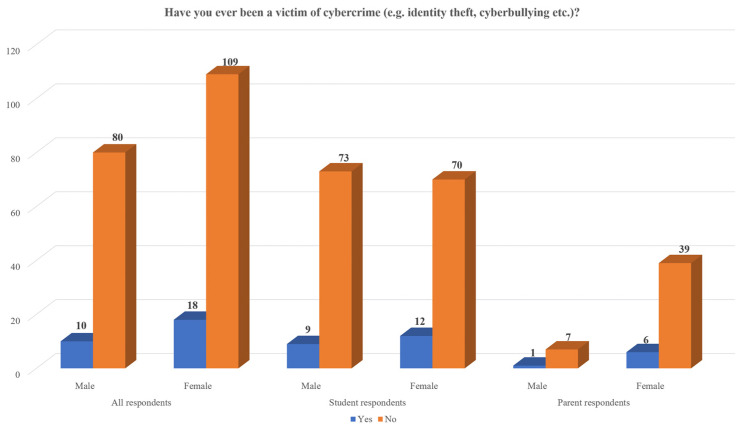
Participants’ responses regarding having been a victim of cybercrime.

Finally, 11.1% of the participants have been a victim of cybercrime (see Figure 2). An almost equal percentage of victims of cybercrime was found between students (12.8%) and parents (13.2%), which points out the significance of this issue among all age groups. In both cases, slightly more female participants reported that they were a victim of cybercrime when compared to their male counterparts. Specifically, among female participants, 14.6% of students and 13.3% of parents had become a cybercrime victim whereas among male participants, 11.0% of students and 12.5% of parents confirmed that they had been a cybercrime victim.

**Figure 3:**
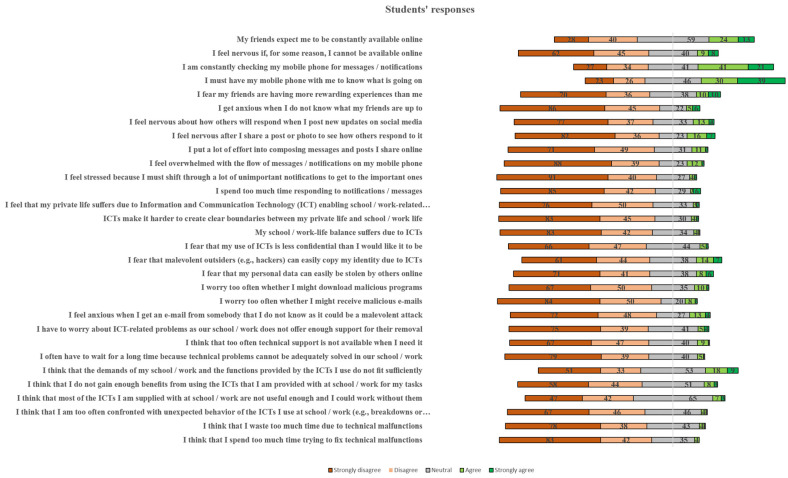
Distribution of students’ responses.

The students’ responses to the 30 Likert scale questions are showcased in Figure 3 in more detail. The “*Availability stress /online vigilance*” stress factor (Q1, Q2, Q3) is the most conspicuous factor (with a mean value of 2.60), followed by the “*FoMO/Fear of Missing Out*” stress factor (Q4, Q5, Q6 with a mean value of 2.37) and the “*Usefulness*” stress factor (Q25, Q26, Q27 with a mean value of 2.25). “*Privacy Intrusion*” stress factor (Q16, Q17, Q18 with a mean value of 2.04), and “*Approval Anxiety*” stress factor (Q7, Q8, Q9 with a mean value of 1.95).

**Figure 4:**
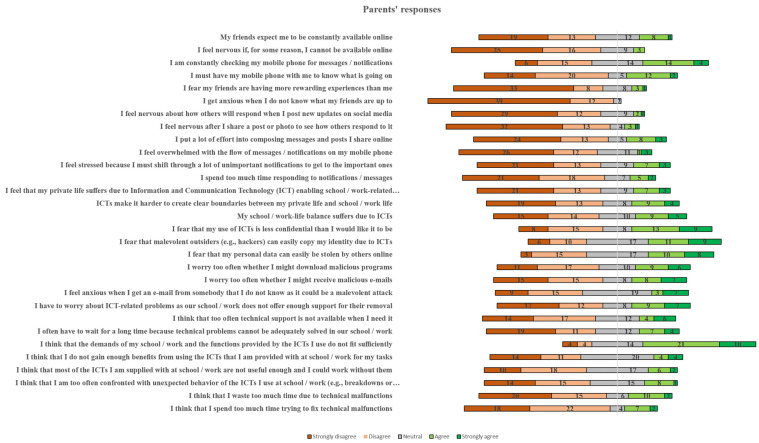
Distribution of parents’ response.

The parents’ responses to the 30 Likert scale questions are showcased in Figure 4 in more detail. The “*Privacy Intrusion*” stress factor (Q16, Q17, Q18) is the most conspicuous factor (with a mean value of 3.07), followed by “*Availability stress/online vigilance*” stress factor (Q1, Q2, Q3 with a mean value of 2.96), “*Usefulness*” stress factor (Q25, Q26, Q27 with a mean value of 2.84), “*Safety/Security*” stress factor (Q19, Q20, Q21) with a mean value of 2.64 and “*Technical* Support” stress factor (Q22, Q23, Q24) with a mean value of 2.46.

Regarding “*Usefulness*”, mixed opinions were expressed about whether the demands of their school/work and the functions provided by the ICTs they use fit. Additionally, it can be said that parents are particularly concerned about the fit between the demands of school/work and the functions provided by the ICTs they use. The mean value of parents’ responses to Q25 is 3.55 and of students’ responses is 2.40. This fact highlights that parents who responded to the questionnaire have good ICT knowledge, and 82.0% of Finns have basic or above basic digital skills in 2023 [45] and consider that the digital tools they use at work do not cover the demands of work. This may also influence how students perceive the tools they use for completing school work. The mean values of the students’ responses clearly give the impression that the most significant digital stress factors are related to how the adolescent appears to peers on social media (availability, approval, and FoMO). They question the usefulness of ICTs they use at school and their own ability to protect their privacy.

When looking at students’ (Figure 3) and parents’ (Figure 4) responses separately, some differences can be observed. For example, Q25 “*I think that the demands of my school/work and the functions provided by the ICTs I use do not fit sufficiently*” has a high value (3.55) for parents, while for students the value is considerably lower (2.40).

Additionally, using an Independent Sample T-test, the data were further examined. Table 5 presents the results of the T-test according to the participants’ status (e.g., students or parents). Specifically, statistically significant differences between students and parents were found in 22 items, as can be seen in Table 5. Questions Q25, Q18, Q16, Q17, and Q20 had the highest difference between students and parents. In all these cases, parents’ ratings were higher than those of students. However, students’ ratings were higher in questions Q4, Q1, Q6, and Q5. Based on the outcomes, it can be stated that the differences in stress factor mean values with students having higher mean values than parents in “*Availability Stress—Online Vigilance*” (1.74) and “FoMO” (0.57). Parents had higher mean values than students in the following stress factors in descending order, “*Privacy Intrusion*” (1.03), “Usefulness” (0.77), “*Safety/Security*” (0.76), *“Conflicts (Blurred boundaries*)” (0.59) and “*Technical Support*” (0.55), and “*Unreliability*” (0.39). Yang and Smith [46] found similar results and, in particular, they emphasized that negative self-perception had a stronger correlation to digital stress than did positive self-perception.

Furthermore, the influence of the participants’ gender was also examined. The related outcomes are presented in Table 6. The biggest differences were found in questions Q18, Q17, Q19, Q3, and Q16. The results show that the differences in digital stressors mean values between female and male responses are, in all cases, higher for females than for males in descending order as follows: “*Privacy Intrusion*” (1.57), “*Connection (Information) Overload*” (1.06), “*Availability Stress/Online Vigilance*” (0.64), “*Approval Anxiety”* (0.62), “*FoMO/Fear of Missing Out*” (0.50), “*Safety/Security*” (0.47), “*Technical Support*” (0.34), and “*Unreliability*” (0.38). The results are in line with the results of Carcelén-García [47] who found that females experienced more negative emotions (social pressure, insecurity and anxiety) than males. Based on the findings, and confirmed by the findings of Carcelén-García [47], it can be said that females are more exposed to online risks than males and also seem to be more vulnerable to digital stress factors. Furthermore, the hours the participants spent on their digital devices daily influenced their responses. The digital stress factors that stand out for students are “*FoMO/Fear of Missing Out*”, “*Availability Stress/Online Vigilance*”, “*Approval Anxiety*”, “*Privacy Intrusion*” and “*Conflicts (Blurred boundaries)*”, whilst for parents, “*Conflicts (Blurred boundaries)*” stands out as significant stress factor.

Parents consider to a higher degree than students that (i) technical support too often is not available when they need it, (ii) they are afraid that their personal online data can easily be stolen by others, and (iii) they do not feel as confident in using ICTs as they would like to. These three main differences in mean values between students and parents show that adolescent students are more confident with using ICTs, they solve their technical problems to a higher degree by themselves and they are also more confident that their personal data will not be stolen, either because they know how to protect themselves or they are ignorant of the danger.

## 5. Discussion

Adolescence is a time period in life with strong emotional, behavioral, cognitive and social changes. This, in turn, coincides with amplified vulnerability to influences from peers and social media influencers and is likely to impact psychological well-being. Studies have found correlations between social media use and psychological problems [48,49], such as anxiety [50], depression [48,51], and reduced self-esteem [52]. Our study confirmed that adolescents are vulnerable to social media as regards how they appear to friends and peers on social media. They need to be constantly available, they are vulnerable to approval regarding their behavior and appearance on social media and they need to constantly check what is going on so that they do not miss out on anything.

When comparing our results to other studies, we conclude that our results are consistent with existing literature on digital stress. For example, Steele et al. [3] created a conceptual model of digital stress including “*Availability stress*”, “*Approval Anxiety*”, “*FoMo*”, and “*Communication Overload*”. Similarly, Khetawat and Steele [15] examined the stress factors “*Availability Stress*”, “*Online Vigilance*”, “*FoMO*”, “*Approval Anxiety*”, and “*Connection (Information) Overload*” and found a significant medium correlation among all of them and psychosocial distress. It is worth noting that students rated “*Availability Stress/Online Vigilance*”, “*FoMO*” and “*Usefulness*” as the three highest digital-stress-evoking factors, while parents regarded “*Privacy Intrusion*”, “*Availability Stress/Online Vigilance*” and “*Usefulness*” as the most significant ones. Therefore, it can be inferred that “*Availability Stress /Online Vigilance*” is a particularly important stress factor both for students and parents and is in line with the results of other studies [3,16,28]. Moreover, Halfmann and Rieger [28] found that “*Availability Stress/Online Vigilance*” is a significant stress factor that creates social pressure. They carried out two studies (N = 61 and N = 197 student participants). Their findings showed that due to the “*Availability Stress/Online Vigilance*” stress factor, social pressure negatively influences autonomy, self-control, and competence. Reinecke et al. [19] (p. 1) developed and validated the Online Vigilance Scale (OVS) that provides a “*new measure of the individual cognitive orientation towards ubiquitous online communication*”. They analyzed three dimensions of online vigilance, namely salience (orientation and attention to the online environment in everyday life), reactibility (tendency to respond to cues from the online sphere), and monitoring (regularly entering the online sphere). They carried out three studies on German smartphone users by using a 5-point Likert scale, and the average mean value for the three studies was 2.55. Our mean value was 2.60. Hence, we conclude that our results regarding “*Availability Stress/Online Vigilance*” are in line with and further validate those of Reinecke et al. [19].

Furthermore, students rated “*FoMO*” as the second highest digital-stress-evoking factor, while parents rated it as the lowest digital-stress-evoking factor. Based on the outcomes, adolescents consider it important to know what their peers are up to. Additionally, when compared to their parents, adolescents are more worried about missing out on interesting things and events. Gugushvili et al. [34] suggested that “*FoMO*” gives rise to excessive checking of social media to know what is happening. They also suggest that “*FoMO*” is negatively associated with compulsive behavior and decreased emotional well-being. Khetawat and Steele [15] emphasized that “*FoMO*” is related to a reduced sense of belonging, self-esteem, and self-control.

Nick et al. [12] emphasized that adolescents feel “*digital stress*” due to the demands of social media, such as worries about peer approval. “*Approval Anxiety*” is a natural feeling in adolescents who are in a phase of identity development and hence, are vulnerable regarding peer approval/disapproval. According to the outcomes, female participants had a higher rate of “*Approval Anxiety*” than male participants, which can be understood from a vulnerability-stress perspective as described in Hallers-Haalboom et al. [53] since females have a higher biologic/psychologic vulnerability toward anxiety. Also, many societies, such as patriarchal societies, have historically forced role models on females which might manifest today into approval anxiety.

In contrast to the results of Khetawat and Steele [15] and Steele et al. [3], no strong evidence of digital stress resulting from “*Connection (Information) Overload*” was found. The students rated it as the least important digital stress factor and the parents as the 8th (out of 10). In their systematic literature review of information overload, Arnolds et al. [54] (p. 6) emphasized that “*the subjective assessment of the quantity of information may be influenced by the available resources and the individual’s ability to manage the incoming information. The quality of information includes the various aspects that contribute to the fit of the information to the needs of the person receiving it. These aspects include, for example, the complexity or relevance of the information*”. They claimed that on a personal level, information overload can be addressed through education. Considering Finnish ICT education, the Finnish Ministry of Education and Culture introduced a digitalization project named the “*digital leap*” in 2015, for modernizing their ICT infrastructure and pedagogy [55]. Today, Finland has “*one of the most advanced and expansive applications of digital technology in education, starting from the first grade of primary school throughout the education system, and consisting of formal as well as extracurricular learning through technology*” [56]. On the same note as Arnolds et al. [54] and considering the high Finnish ICT education, our outcomes highlight that selecting and managing potentially useful information (instead of letting the load of information become a hindrance) is something that can be learned. Hence, our results further confirm the applicability of this theory. Continuing on the importance of education and cognitive skills, our study showed that both students and parents rated the usefulness of ICTs that they use at school/work and the confidence they have in using ICTs for protecting their privacy as a high digital stress factor (third most important). Bali et al. [57] emphasized that positive cognitive functions (i.e., gained through education) predict better computer and smartphone skills which also reduce technostress (stress induced by ICTs), increase positive attitudes toward ICT use, and enhance confidence in ICT learning. However, the reasons why the Finnish respondents in this study did not consider “*Connection (Information) Overload*” to be a stress factor need to be investigated in more detail.

We added the expression “*blurred boundaries*” to the stressor category “*Conflicts*” coined by Fisher et al. [40] because the conflicts refer to the difficulties of separating real life from the digital life/world of social media. Back in 2012, Sánchez Abril et al. [58] used the expression “*blurred boundaries*” for courts that were struggling to separate what is professional (work) communication and what is private communication. Palm et al. [59] emphasized that stress in the working environment is not only related to work and stress in the private environment is not only related to private life. The boundaries are blurred. The study also showed that this kind of stress is usually sporadic. Adolescents in our study rated the digital stress factor “*Conflicts/Blurred Boundaries*” as the 9th (out of 10) most important factor while the parents rated it as the 6th. This shows that students likely are able to separate school life from private life to a higher degree, eventually because of strict rules regarding schools’ communication outside school hours. This may also be attributed to the wide-ranging ICT education Finnish students receive already from a very young age.

Moreover, parents rated “*Privacy Intrusion*” as the highest digital stress factor, whilst students rated it as the fourth highest digital stress factor. Parents and students are anxious about all collected and shared personal information and the “*Safety/Security*” of this information. We added the word security to the stressor category “*Safety*” coined by Fisher et al. [40] because this question deals with cyber security and unauthorized access. “*Safety/Security*” was rated at fourth place by the parents and seventh by the students. “*Privacy Intrusion*” is a significant stress factor and can be, when misused, a legal concept regarding intrusion into the private life of a person. The level of potential privacy intrusion perception can even impact engagement and participation in social media platforms [60,61,62]. This is further validated in the qualitative study of Winstone et al. [63] in which the participants instinctively raised concerns of privacy regarding disclosure of personal information and social media broadcasting.

The total digital stress perception experienced by an individual can be expressed as the average across all 30 questions [38]. The higher the value, the more noticeable the perceived digital stress is. The total digital stress perception experienced by students was 2.04 and by parents was 2.43. This shows that the parents have slightly higher total digital stress perception than the students. Both values are relatively low (Likert scale 1–5) which shows that the respondents are able to cope with digital stressors.

## 6. Conclusions

This paper contributes to debates regarding the effect of the use of digital resources, ICTs, and social media on processes at school/work and at home. The findings of our study confirmed earlier findings about “*Availability Stress/Online Vigilance*”, “*FoMO*”, and “*Approval Anxiety*”, but regarding “*Connection (Information) Overload*”, our results showed that it is not an important digital stress factor.

Further work will concentrate on a qualitative study of the “*Connection (Information) Overload*” and the reasons why Finnish students do not find it as a stress factor. Future work will also focus on carrying out case studies in different countries and different levels of education regarding digital stress and overload as well as identifying potential solutions for alleviating the negative impacts of digital technologies on students and parents. The results of this study will also be the basis for a qualitative follow-up study, in which interviews, operations, and discussions will be conducted to acquire more in-depth viewpoints and experiences of different education stakeholders. Future research will focus on examining the impact and relationship among the different stress factors as well as the importance and influence of resilience. Finally, after having further explored the phenomenon of digital stress in a generalized context, a study regarding a construct of coping mechanisms and adolescent digital stress resilience will be carried out.

Understanding the characteristics of digital stress is of utmost importance to finding suitable coping mechanisms and facilitating resilience toward digital stress. A practical implication for future research directions could be the development of a mobile application that utilizes a smartphone in the daily activity of a person for monitoring critical signs of human autonomic function. We propose further research into artificial intelligence-based systems that are capable of tracking and monitoring human digital stress and resilience. Such systems have the potential to aid in the development and activation of coping mechanisms.

However, the study is characterized by some limitations that should be noted. Specifically, the limitations in this study relate to the use of a single data collection method in one particular population (a secondary school in Finland). Multiple data collection methods (e.g., Triangulation) would improve the research strategy for testing validity through the convergence of information from diverse sources. To generalize the results, a research sample from a wider population would minimize cultural and environmental bias. Finally, the lack of a network analysis of the data can be regarded as another limitation of this study.

To sum up, this study revealed that the most important stress factors for parents and students were “Privacy Intrusion”, “Availability stress/Online vigilance”, and “Usefulness”. When examining the data of students and parents separately, the “Fear of Missing Out/FoMO” and “Approval Anxiety” were the most influential stress factors for students while “Safety/Security” and “Technical Support” factors were the most impactful stress factors for parents. When assessing the overall digital stress of students and parents, no significant differences were observed. Finally, in contrast to the existing literature, no connection between “Connection (Information) Overload” and digital stress was found.

## Figures and Tables

**Table 1:** Digital-stress-evoking factors [39] (p. 422).

Public Shaming and Humiliation	*“Humiliating, broadcasted messages, often in the form of insults, posted on social media or forwarding of nude pictures to unintended audiences*”.
Impersonation	“*Masking an individual’s identity and pretending to be someone else, generally for the purpose of insulting, mocking or embarrassing the impersonated*”.
Harassing and Personal Attacks	“*Directly receiving unwanted messages and personal attacks through digital devices or accounts*”.
Breaking and Entering	“*Logging into another person’s online accounts or looking through their digital devices without permission*”.
Pressure to comply	“*Managing requests (generally unwanted) to grant access to accounts or nude photographs to close others*”.
Smothering	“*Constant messaging or contact, not intended to hurt nor harm, but the quantity is itself problematic*”

**Table 2:** Questionnaire structure.

Stress Factor	Questions
Availability Stress/Online Vigilance	Q1. My friends expect me to be constantly available online.
	Q2. I feel nervous if, for some reason, I cannot be available online.
	Q3. I am constantly on my phone for messages/notifications.
FoMO/Fear of Missing Out	Q4. I must have my phone with me to know what is going on.
	Q5. I fear my friends are having more rewarding experiences than me.
	Q6. I get anxious when I don’t know what my friends are up to.
Approval Anxiety	Q7. I feel nervous about how others will respond when I post new updates on social media.
	Q8. I feel nervous after I share a post or photo to see how others respond to it.
	Q9. I put a lot of effort into composing messages and posts I share online.
Connection (Information) Overload	Q10. I feel overwhelmed with the flow of messages/notifications on my phone.
	Q11. I feel stressed because I must sift through a lot of unimportant notifications to get to the important ones.
	Q12. I spend too much time responding to notifications/messages.
Conflicts (Blurred boundaries)	Q13. I feel that my private life suffers due to Information and Communication Technology (ICT) enabling school/work-related problems to reach me everywhere.
	Q14. ICTs make it harder to create clear boundaries between my private life and school/work life.
	Q15. My school/work-life balance suffers due to Information and Communication Technologies (ICTs).
Privacy Intrusion	Q16. I fear that my use of ICTs is less confidential than I would like to.
	Q17. I fear that malevolent outsiders (e.g., hackers) can easily copy my identity due to ICTs.
	Q18. I fear that my personal data can easily be stolen by others online.
Safety/Security	Q19. I have to worry too often, whether I might download malicious programs.
	Q20. I have to worry too often, whether I might receive malicious e-mails.
	Q21. I feel anxious when I get an e-mail from somebody that I do not know as it could be a malevolent attack
Technical Support	Q22. I have to worry about ICT-related problems as our school/work does not offer enough support for their removal.
	Q23. I think that it happens too often that technical support is not available when I need it.
	Q24. I often have to wait for a long time because technical problems cannot be adequately solved in our school/work.
Usefulness	Q25. I think that the demands of my school/work and the functions provided by the ICTs I use do not fit sufficiently.
	Q26. I think that I do not gain enough benefits from using the ICTs that I am provided with at school/work for my tasks.
	Q27. I think that most of the ICTs I am supplied with at school/work is not useful enough and I could work without them.
Unreliability	Q28. I think that I am too often confronted with unexpected behavior of the ICT I use at school/work (e.g., breakdowns or long response times).
	Q29. I think that I lose too much time due to technical malfunctions.
	Q30. I think that I spend too much time trying to fix technical malfunctions.

**Table 3:** Average number of hours spent on digital devices per day.

	All		Students		Parents	
	Freq.	Pct.	Freq.	Pct.	Freq.	Pct.
0–1 h	11	5.1	11	6.7	0	0
2–5 h	160	73.7	121	73.8	39	73.6
6–10 h	43	19.8	31	18.9	12	22.6
More than 10 h	3	1.4	1	0.6	2	3.8

**Table 4:** Parents’ work experience in years.

	Parents	
	Freq.	Pct.
0–5 years	6	11.3
6–10 years	9	17.0
11–20 years	18	34.0
21–30 years	17	32.1
More than 30 years	3	5.70

**Table 5:** Questions with significant differences in response between students and parents.

	Student Mean Value	Parent Mean Value	F	Sig.	t	df	p
Q1	2.72	2.23	0.267	0.606	2.714	215	0.007
Q4	3.22	2.40	0.747	0.388	3.957	215	0.000
Q5	2.11	1.70	1.514	0.220	2.231	215	0.027
Q6	1.78	1.30	17.504	0.000	4.366	171.332	0.000
Q11	1.70	2.21	11.381	0.001	−2.734	70.583	0.008
Q13	1.81	2.21	12.732	0.000	−2.120	70.075	0.038
Q14	1.76	2.36	19.815	0.000	−3.035	68.732	0.003
Q15	1.77	2.53	20.719	0.000	−3.895	68.343	0.000
Q16	1.96	3.00	18.440	0.000	−5.160	69.259	0.000
Q17	2.16	3.13	0.208	0.649	−5.269	215	0.000
Q18	2.01	3.09	0.169	0.681	−6.221	215	0.000
Q19	1.96	2.66	11.360	0.001	−3.582	72.507	0.001
Q20	1.74	2.57	23.718	0.000	−4.011	67.853	0.000
Q21	1.96	2.70	1.516	0.220	−4.232	215	0.000
Q22	1.93	2.57	19.345	0.000	−3.005	69.951	0.004
Q23	1.96	2.45	8.317	0.004	−2.559	72.096	0.013
Q24	1.84	2.36	14.601	0.000	−2.674	70.329	0.009
Q25	2.40	3.55	1.583	0.210	−6.204	215	0.000
Q26	2.11	2.49	2.652	0.105	−2.285	215	0.023
Q28	1.94	2.38	4.628	0.033	−2.629	76.999	0.010
Q29	1.85	2.23	8.863	0.003	−1.998	71.747	0.050
Q30	1.76	2.11	1.083	0.299	−2.394	215	0.018

**Table 6:** Questions with significant response differences between male and female participants.

	Male Mean Value	Female Mean Value	F	Sig.	t	df	p
Q3	2.59	3.21	0.415	0.520	−3.739	215	0.000
Q6	1.50	1.78	6.590	0.011	−2.279	214.305	0.024
Q7	1.62	2.12	8.564	0.004	−3.601	214.799	0.000
Q8	1.58	2.10	9.952	0.002	−3.571	214.832	0.000
Q11	1.61	1.97	2.203	0.139	−2.545	215	0.012
Q12	1.61	2.02	2.917	0.089	−2.905	215	0.004
Q15	1.76	2.09	1.827	0.178	−2.321	215	0.021
Q16	1.88	2.46	5.536	0.020	−3.889	210.912	0.000
Q17	1.98	2.69	13.844	0.000	−4.576	214.425	0.000
Q18	1.82	2.59	6.792	0.010	−5.089	212.599	0.000
Q19	1.73	2.42	16.529	0.000	−4.979	214.659	0.000
Q20	1.62	2.17	14.117	0.000	−3.931	213.726	0.000
Q21	1.90	2.31	7.569	0.006	−2.695	210.954	0.008
Q22	1.84	2.25	2.498	0.115	−2.565	215	0.011
Q23	1.81	2.28	8.445	0.004	−3.388	214.532	0.001
Q25	2.37	2.90	2.029	0.156	−3.089	215	0.002
Q27	2.09	2.46	0.886	0.348	−2.709	215	0.007

## Data Availability

The data supporting the conclusions of this article will be made available by the corresponding authors on request.
